# Mineral Phosphorus Supply in Piglets Impacts the Microbial Composition and Phytate Utilization in the Large Intestine

**DOI:** 10.3390/microorganisms9061197

**Published:** 2021-06-01

**Authors:** Henry Reyer, Per J. R. Sjöberg, Michael Oster, Aisanjiang Wubuli, Eduard Murani, Siriluck Ponsuksili, Petra Wolf, Klaus Wimmers

**Affiliations:** 1 Leibniz Institute for Farm Animal Biology (FBN), Institute for Genome Biology, Wilhelm-Stahl-Allee 2, 18196 Dummerstorf, Germany; reyer@fbn-dummerstorf.de (H.R.); oster@fbn-dummerstorf.de (M.O.); wubuli@fbn-dummerstorf.de (A.W.); murani@fbn-dummerstorf.de (E.M.); ponsuksili@fbn-dummerstorf.de (S.P.); 2 Department of Chemistry-BMC, Uppsala University, 75123 Uppsala, Sweden; Per.Sjoberg@kemi.uu.se; 3 Nutrition Physiology and Animal Nutrition, Faculty of Agricultural and Environmental Sciences, University of Rostock, 18059 Rostock, Germany; petra.wolf@uni-rostock.de; 4 Animal Breeding and Genetics, Faculty of Agricultural and Environmental Sciences, University of Rostock, 18059 Rostock, Germany

**Keywords:** phosphorus, inositolphosphate, large intestine, 16S rRNA, pigs

## Abstract

A sufficient supply of phosphorus (P) to pigs in livestock farming is based on the optimal use of plant-based phytate and mineral P supplements to ensure proper growth processes and bone stability. However, a high P supplementation might bear the risk of higher environmental burden due to the occurrence of excess P and phytate degradation products in manure. In this context, the intestinal microbiota is of central importance to increase P solubility, to employ non-mineral P by the enzymatic degradation of phytate, and to metabolize residual P. A feeding experiment was conducted in which piglets were fed diets with different P levels, resulting in three groups with low, medium (covering requirements), and high concentrations of available P. Samples from caecum and colon digesta were analysed for microbial composition and phytate breakdown to estimate the microbial contribution to metabolize P sources. In terms of identified operational taxonomic units (OTU), caecum and colon digesta under the three feeding schemes mainly overlap in their core microbiome. Nevertheless, different microbial families correlate with increased dietary P supply. Specifically, microbes of *Desulfovibrionaceae, Pasteurellaceae*, *Anaerovoracaceae*, and *Methanobacteriaceae* were found significantly differentially abundant in the large intestine across the dietary treatments. Moreover, members of the families *Veillonellaceae, Selenomonadaceae*, and *Succinivibrionaceae* might contribute to the observed phytate degradation in animals fed a low P diet. In this sense, the targeted manipulation of the intestinal microbiota by feeding measures offers possibilities for the optimization of intestinal phytate and P utilization.

## 1. Introduction

The viability of all organisms depends on a demand-covering supply of phosphorus (P) for the establishment of structural body compartments and the maintenance of other important physiological functions, such as energy supply, cell signalling, and blood buffering. For pigs, the main P sources are phosphorus-containing mineral supplementations (dicalcium-/monocalcium-phosphates) produced from rock phosphates and also phytic acid (*myo*-inositol 1,2,3,4,5,6-hexakis; InsP6), which is usually present as phytate and represents the storage form of P in plants [1,2]. The provision of P available to the animal from phytate is essential to meet the needs of the organism while using a sustainable mineral source. However, the phytate utilization requires its gradual hydrolysis, which is catalysed by phytases and other phosphatases, such as the intestinal alkaline phosphatase. Depending on dietary supply and gastrointestinal site, the cleavage steps result in variable amounts of phosphate, inositolphosphates (InsPx), and *myo*-inositol. However, due to the low impact of endogenous intestinal phosphatases on phytate hydrolysis, pigs have a low capacity to utilise phytic acid, which requires the involvement of phytases of plant or microbial origin [3]. In practice, exogenous phytases of microbial origin are added to pig feed, improving the available P content based on the amount and origin of phytases [2]. However, considering the microbial community of the gastrointestinal tract of pigs and chickens, it is known that some of the microbes present are able to produce phytase and secrete them into the lumen. These include *Bifidobacteria* [4], various isolates of *Lactobacillus* [5,6], and *Pediococcus* [7] as well as *Pseudomonas* spp. [8]. This exemplifies the potentially broad microbial capacity to degrade phytate and provide P in the gastrointestinal tract of monogastric animal species. Besides the benefits of phytate as a P source, antinutritive effects of InsP6 (inositol hexakisphosphate), InsP5 (inositol pentakisphosphate), InsP4 (inositol tetrakisphosphate), and InsP3 (inositol triphosphate) have been described. These include mainly the impairment of the protein-degradation capacity of pepsin and the drastic reduction of the solubility of zinc at pH values relevant for the proximal small intestine in the presence of InsPx [2,9]. Therefore, an efficient breakdown of phytate in the gastrointestinal tract is preferable. Moreover, there is an interaction of dietary mineral P and InsPx such that high P hampers the degradation of phytase [10]. At present, several approaches proved beneficial for the rapid hydrolysis of phytate, e.g., liquid feeding, phytase superdosing, or feed pre-treatment [11,12]. Considering the above-mentioned potential of microbes present in the gastrointestinal tract to provide phytases and improve P efficiency, dietary strategies might further include the establishment of a favourable intestinal microbiome profile. Shifts in the microbial composition of the intestine will facilitate meeting the required P supply by exploiting a higher degree of non-mineral P of plant origin to reduce P excretion and thus to reduce the environmental impact. Therefore, the current study focuses on the investigation of the microbial composition and the profile of inositolphophates in the pig large intestine in response to varying dietary P intakes.

## 2. Materials and Methods

### 2.1. Animal Trial and Sample Preparation

The animal experiment to which this study refers was approved by the ethics committee of the federal state of Mecklenburg-Western Pomerania, Germany (Landesamt für Landwirtschaft, Lebensmittelsicherheit und Fischerei; LALLF-M-V/TSD/7221.3-1-053-15). A feeding trial was carried out in an experimental pig house with varying levels of mineral P fed to piglets between 28 and 64 days of life, as previously described [13]. In brief, dietary groups were composed as follows: group L consisting of 8 piglets fed with a lower mineral P level (P = 0.6% of DM), group M consisting of 6 piglets fed recommended P levels (P = 0.9% of DM), and group H consisting of 7 piglets fed with a higher mineral P level (P = 1.1% of DM). Except P, all other nutrient contents of the feed were based on current recommendations [14]. The diets had comparable levels of protein (L: 205 g/kg DM; M: 203 g/kg DM; H: 201 g/kg DM), metabolizable energy (L: 12.5 MJ/kg DM; M: 12.5 MJ/kg DM; H: 12.3 MJ/kg DM), and calcium (L: 13.0 g/kg DM; M: 13.0 g/kg DM; H: 13.0 g/kg DM) [13]. Vitamin D3 was provided at the level of 1000 IU for all dietary groups. No microbial phytases were added to the wheat/barley/soybean meal-based diets. The piglets originated from 4 litters and were equally assigned to dietary groups with at least 3 males and females per group. The trial was carried out in March–April at ambient stable temperature and relative humidity. A constant 12-h light-dark cycle was applied. The piglets were kept individually on a flat-deck and had ad libitum access to pelleted feed and water [13]. At the end of the trial period, pigs were killed by exsanguination following electric stunning. From all pigs, digesta samples were collected from the terminal tip of the caecum and the mid region of the colon. Subsequently, samples were shock frozen in liquid nitrogen and stored at −80 °C until microbiota and inositolphosphate analyses.

### 2.2. 16S rRNA Profiling

DNA extraction of pig digesta samples followed the instructions of the PowerLyzer PowerSoil DNA Isolation Kit (MoBio, Carlsbad, CA, USA) with additional heating steps of 10 min at 70 °C and 95 °C prior to beat beating. Amplificates of the 16S rRNA gene were produced in duplicates using primers specific for variable region V4 (515’F and 806R), including adapters and barcodes [15,16]. Polymerase chain reactions were performed with 5PRIME HotMasterMix (5 PRIME, Hamburg, Germany) as follow: 95 °C for 2 min, 30 cycles at 95 °C for 30 s, 55 °C for 60 s, 72 °C for 90 s, and a final extension for 10 min at 72 °C. PCR products were purified using magnetic beads (Agencourt AMPure XP, Beckman Coulter, Krefeld, Germany) and mixed in equal concentrations. Sequencing was performed on a HiSeq2500 (Illumina, San Diego, CA, USA) generating 250 bp paired-end reads. After demultiplexing of the sequencing reads, raw data were analysed with the mothur software (version 1.44.1) [17]. Sequences were globally aligned to the Silva reference database (release 138) with chimeric sequences removed. Considering a sequence identity of 97%, the sequences were combined into operational taxonomic units (OTU), and OTU annotations were retrieved from the Silva database (release 138).

### 2.3. Inositolphosphate Analysis

For the analysis of InsP6 and InsP5, approximately 200 mg of digesta samples obtained from caecum and colon were lyophilized. The chemicals used were of analytical quality (Sigma-Aldrich, Taufkirchen, Germany). In-house stock solution was prepared as previously described [18,19] from the dipotassium salt of myo-inositol hexakis (dihydrogenphosphate) (P5681, Sigma-Aldrich, Taufkirchen, Germany). A standard series was prepared by diluting the in-house stock solution with 0.1 M NaOH containing 0.01 M etylenediamin tetraacetic acid (EDTA) in the range between 0.1–55 µM for calibration of the ESI-MS system. Freeze-dried samples (approx. 20 mg) were extracted with 1 mL 1.0 M NaOH containing 0.1 M EDTA. Samples were shaken with a Multi Reax for 16 h (Heidolph Instrument, Schwabach, Germany). To remove particles, the samples were centrifuged at 10,000 rpm for 15 min. The supernatant (0.1 mL) was mixed with 0.9 mL of Milli-Q water in a glass vial before placing it in the LC autosampler for analysis. The 1260 Infinity (Agilent Technologies, Waldbronn, Germany) chromatographic system was used in connection with a 3200 Q Trap LC/MS/MS system (AB Sciex, Concord, ON, Canada). The LC-MS method was the same as previously reported [19]. InsP6 and InsP5 were detected by using multiple reaction monitoring mode (MRM) with suitable precursor and product ion transitions with optimal collision energy (CE) and collision cell exit potential (CXP). These values were evaluated in a previous work [19]. Data acquisition and quantification were carried out with Analyst 1.4.2 (AB Sciex). All MRM transitions for respective InsPx were summed before manual peak integration.

### 2.4. Data Analysis

The OTU abundances determined were adjusted by means of subsampling, taking into account the library with the fewest sequences. After taxonomic annotation of OTU, the relative abundance of phyla and family level were visualized in taxa plots employing the R software. Dietary differences at family level were assessed separately for caecum and colon using DESeq2 (DOI:10.18129/B9.bioc.DESeq2). Therefore, very low abundant families were excluded by considering only taxa with more than ten observations in at least 30% of all samples. A likelihood ratio test was performed against a base model with mother and sex of piglets as effects. Differences were considered significant at a Benjamini–Hochberg adjusted *p*-value < 0.05. Data referring to the InsPx levels in caecum and colon contents were summarized as mean ± SE. One sample from the L group had to be removed from the analyses due to missing values. Data were analysed in a linear model (R package lmerTest, v3.1-2) considering effects of dietary P content, litter, and sex. The significance threshold was set at *p* < 0.05. For correlation analysis between phenotypes and microbiota, Kendall correlation coefficients were calculated. Therefore, animal-individual values of P intake and serum P levels were obtained from a previous study and considered in the analysis [13]. 

## 3. Results and Discussion

**Figure 1:**
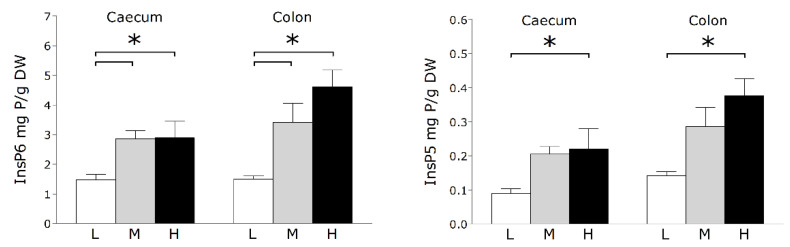
Levels of inositol hexakisphosphate (InsP6) and inositol pentakisphosphate (InsP5) in the caecum and colon of piglets fed a varying supply of mineral P for a period of 5 weeks. Significant differences are indicated by an asterisk (*; *p* < 0.05). L, M, H—dietary groups receiving lower, medium, and higher mineral P levels; DW—dry weight.

The measurement of inositolphosphates in caecum and colon digesta revealed a gradual increase of InsP6 and InsP5 in the two intestinal sections with increasing mineral P supply in the diets (Figure 1). The L group showed significantly lower levels of InsP6 and InsP5 in both intestinal segments compared to M and H (*p* < 0.05), although this difference was not considered significant when comparing InsP5 levels between L and M (*p* = 0.05 for colon, *p* = 0.08 for caecum). Despite the absence of a phytase of exogenous origin in the feed, there is a clear response to reduced mineral P supply in the L group resulting in an increased hydrolysis of phytate. Supporting this observation, it was found that phytase activity in the ileum increased with decreasing P in the diet [20]. The group-specific patterns and magnitude of effects were similar for caecum and colon digesta, although, a higher amount of inositolphosphates was found for H in the colon compared to the caecum (*p* < 0.05 for InsP6 and InsP5). 

**Figure 2:**
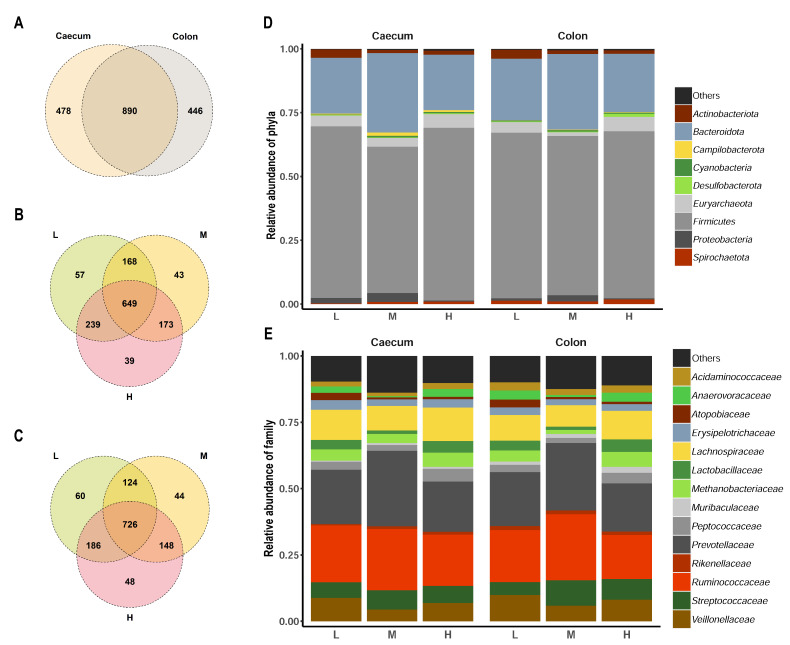
Microbial composition of caecum and colon digesta of pigs fed with varying mineral P levels. Venn diagram representation of specific and overlapping OTUs considering (**A**) caecum and colon digesta samples, (**B**) caecum digesta samples from the three dietary groups, and (**C**) colon digesta samples from the dietary groups. Taxa plots at (**D**) phylum and (**E**) family level with the 9 and 14 top taxa, respectively. L, M, H—dietary groups receiving lower, medium, and higher mineral P levels.

Since the experimental design excluded exogenous phytase supplements, the main factor for the observed differences in an inositolphosphate degradation likely refers to the intestinal microbiota composition. The sequencing of the 16S rRNA revealed that, at OTU level, about two-thirds of the taxonomic units overlapped between caecum and colon digesta (Figure 2A). This corresponds to the previous observation that caecum and colon exhibit a high similarity in microbiota profiles [21]. Regarding the specific dietary groups in both tissues, the relations represented in the Venn diagrams revealed a considerable amount of common OTU interpreted as independent of the dietary P supply (Figure 2B,C). However, 39 to 60 OTUs were identified to be specific for a single dietary group, without a particular group displaying a considerable increase in microbial richness. Interestingly, the overlap in OTUs was numerically higher in the extreme P groups (239 and 186 in caecum and colon, respectively, considering L and H) compared to the contrasts with the M group. This might be related to observations that varying dietary P content and phytase addition are associated with the capacity of the cellular adaptive immune system and influence the metabolism in terms of fibre digestion and the concentration of microbial metabolites [22,23,24].

**Figure 3:**
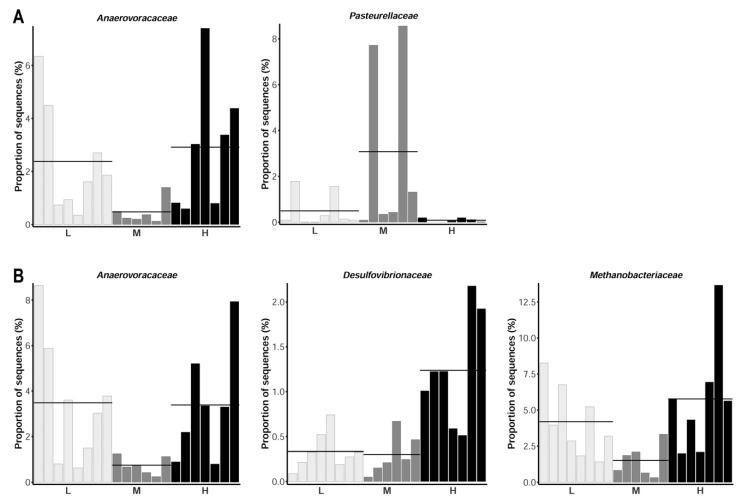
Significant differentially abundant taxa in (**A**) caecum and (**B**) colon digesta at family level (adjusted *p* < 0.05). Individual proportions of sequences assigned to the taxa are represented for each of the three dietary groups. L, M, H—dietary groups receiving lower, medium, and higher mineral P supplements.

Diet-specific taxa plots at phylum level revealed a large overlap in the microbiota composition between the two intestinal segments and the three dietary groups (Figure 2D). The most abundant phyla comprised Firmicutes and Bacteroidota, with an average relative abundance of 0.64 and 0.25, respectively. The abundance of these two predominant phyla reflects the typical microbiota composition in the pig at this age [21]. Group-specific differences in the average abundance were consistent across tissue sites. Considering the top 14 taxa at the family level, *Prevotellaceae* and *Ruminococcaceae* dominated in caecum and colon (Figure 2E). Statistical analysis at family level revealed that microbes assigned to *Anaerovoracaceae* and *Pasteurellaceae* in the caecal digesta were found to be significantly differentially abundant between dietary groups (adjusted *p* < 0.05, Figure 3, Supplementary Tables S1 and S2). As shown in the animal-individual representation of abundances of *Pasteurellaceae* in Figure 3A, the differences were mainly driven by two samples of the M group showing a considerably high proportion of sequences assigned to this taxon. For *Anaerovoracaceae*, samples of L and H groups showed higher abundance compared to M animals. In the colon digesta, *Anaerovoracaceae* were also differentially abundant between the dietary groups on family classification, showing the same diet-specific pattern likewise in caecum. The family *Anaerovoracaceae* is sparsely characterized. It belongs to the class of *Clostridia*, which are typically involved in the fermentation of plant polysaccharides in the gastrointestinal tract [25]. In colon digesta, also *Methanobacteriaceae* and *Desulfovibrionaceae* differed significantly in their abundance between dietary groups. *Methanobacteriaceae* was lowest abundant in M, whereas microbes of *Desulfovibrionaceae* were found predominantly in the colon digesta of H animals. The predominant process for energy generation in *Methanobacteriaceae* is the reduction of carbon dioxide to form methane [26]. However, it has recently been shown that the abundance of *Archaeae*, which include *Methanobacteriaceae*, is correlated to phytase supplementation in pigs [27]. For *Desulfovibrionaceae*, the genus *Desulfovibrio* was found to have a significantly higher relative abundance in the jejunum and caecum of goats with a high digestibility of P [28]. Therefore, independent of the actual functional involvement in P metabolism, representative species of these two families could drive potential improvements of P availability and phytate utilization in the gastrointestinal tract of pigs.

**Figure 4:**
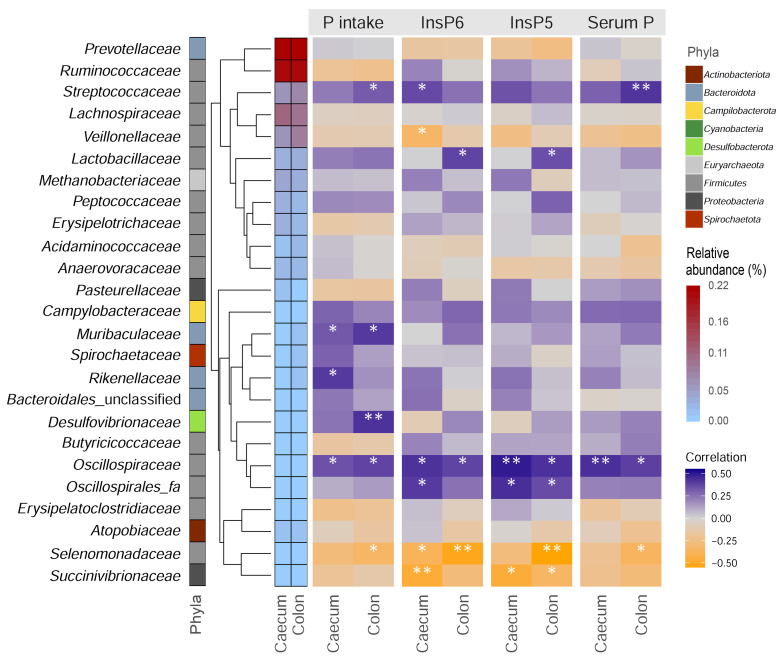
Correlation heatmap. Data of the 25 most abundant microbial families detected in the large intestine were correlated with phosphorus (P) intake, inositol-6 phosphate (InsP6), inositol-5 phosphate (InsP5), and serum P levels. The phyla column indicates the assignment of families to phyla. The cladogram represents the hierarchical clustering of taxa based on their abundance. Correlation coefficients considered significant are indicated by an asterisk (*: *p* < 0.05, **: *p* < 0.01).

The variable dietary P supply provided to the pigs in this study affects various levels of the organism such as the chemical composition and the microstructure of the bones as well as the hormone level and the mineral concentration in the blood to primarily maintain P homeostasis [13]. In order to further explore the interaction between dietary P intake, inositolphosphate degradation, and the abundance of microbial taxa in the caecum and colon, correlation analyses were performed. The majority of the 25 most abundant families included in the analysis originated from *Firmicutes* (Figure 4). With respect to the microbial families with higher relative abundance, significant positive correlation coefficients were identified for *Streptococcaceae* with P intake und serum P levels in colon and with InsP6 values in caecum. This implies favourable conditions for the growth of *Streptococcaceae* under higher available P concentrations in the digesta. It has been described that species of *Streptococcaceae* perform proteolytic processes in the colon and contribute substantially to the turnover of sulfur-containing substrates [29]. Phytase activity has not yet been reported for representatives of this microbial family. *Veillonellaceae* were significantly negatively correlated with InsP6 levels in caecum digesta. This indicates an increased phytate utilization in the presence of microbes of this family. Interestingly, by adding exogenous phytase to the diets of growing pigs, the relative abundance of *Veillonellaceae* significantly decreased [27]. For *Lactobacillaceae*, a positive correlation with InsP6 and InsP5 in colon was retrieved. For low abundant taxa, increasing levels of dietary mineral P intake were suggested to correlate with an increased presence of *Muribaculaceae*, *Rikenellaceae*, and *Desulfovibrionaceae*. For *Muribaculaceae* and *Rikenellaceae*, so far, no information is available about their involvement in P metabolism. Members of the former are known for degradation of carbohydrates and utilization of nitrogen, whereas the latter are mainly hydrogen-producing bacteria [30]. In accordance with the results of the statistical comparison of dietary groups in this study, the identification of *Desulfovibrionaceae* provides clear evidence of a relationship between the dietary intake of mineral P and the occurrence of members of this microbial family. The abundance of *Oscillospiraceae* was significantly positively correlated with P intake, InsP6, and InsP5 abundance and serum P levels in both intestinal segments. These conditions of higher dietary P availability and a higher phytate reservoir could lead to conditions with more available P in the large intestine, which represent favourable conditions for certain microbes. Indeed, members of the *Oscillospiraceae* were previously shown to be increased abundantly due to phytase supplementation [27]. Moreover, other representatives of the order *Oscillospirales*, namely Oscillospirales_fa were positively correlated with caecum InsP6 and InsP5 as well as colon InsP5. *Selenomonadaceae* and *Succinivibrionaceae* showed significant negative correlation coefficients with InsP6 and InsP5 levels in different parts of the large intestine. Further, the abundance of *Selenomonadaceae* in the colon was significantly correlated with P intake and serum P levels. Interestingly, a certain phytase activity was detected in several species belonging to the *Selenomonadaceae*, among which, e.g., *Mitsuokella* were considered to have a high activity [31].

Besides the essential capacity for P absorption in the small intestine, the role of P metabolism in the caecum and colon is still largely unclear. It is known for certain that P is released from phytic acid by hydrolysis in the large intestine of pigs, which in turn is available for use by microbial species [32]. However, there are various indications for an uptake of P into the organism also via the epithelium of the large intestine [23]. Specifically, indications are provided by the post-ileal disappearance of P and the expression of P-transporters in the terminal colon [33,34].

## 4. Conclusions

The current study identifies a number of microbial taxa that either influence the P availability in the large intestine through their phytase activity or are positively or negatively influenced by higher intestinal P levels in terms of growth and replication. Microbial families that might benefit from increasing dietary P supply comprise *Streptococcaceae, Muribaculaceae*, *Rikenellaceae*, *Desulfovibrionaceae*, and *Oscillospiraceae*. Potential to provide additional phytase activity and thus induce the hydrolyses of inositolphosphates might be contributed by members of the families *Veillonellaceae, Selenomonadaceae*, and *Succinivibrionaceae*. A P-reduced diet for pigs triggers microbial-mediated compensatory actions through phytate cleavage and P release, thereby increasing available P to the host and gut microbiota. In addition to changes in the abundance of certain taxa associated with P digestion and inositol degradation, changes in microbial enzyme expression and functional shifts of microbes in response to P-variable diets may also be important and can be revealed by metatranscriptome analysis.

## Supplementary Materials

The following are available online at https://www.mdpi.com/article/10.3390/microorganisms9061197/s1, Table S1: Group-specific proportions of sequences assigned to taxa at family level in the caecum digesta; Table S2: Group-specific proportions of sequences assigned to taxa at family level in the colon digesta.
